# The effect of alveolar recruitment maneuver on intraoperative hemodynamics: A randomized controlled trial

**DOI:** 10.1097/MD.0000000000050848

**Published:** 2026-09-25

**Authors:** Hasan Duman, Ebru Duman, Hafize Fisun Demir, Fatih Ugun, Nazan Kocaoglu Bugra, Serkan Uckun, Ozlem Sagir

**Affiliations:** a Clinic of Anesthesiology and Reanimation, Balikesir Atatürk City Hospital, Balikesir, Turkey; b Clinic of Anesthesiology and Reanimation, Zonguldak Alapli State Hospital, Zonguldak, Turkey; c Department of Anesthesiology and Reanimation, Balikesir University Medical Faculty, Balikesir, Turkey.

**Keywords:** alveolar recruitment maneuver, compliance, hemodynamics, laparoscopic surgery, oxygenation

## Abstract

**Background:**

Pneumoperitoneum and the Trendelenburg position during laparoscopic hysterectomy (LH) increase intra-abdominal and intrathoracic pressure, reducing lung compliance and predisposing patients to atelectasis. This study evaluated the effects of alveolar recruitment maneuver (RM) on intraoperative hemodynamics, lung compliance, and oxygenation.

**Methods:**

Following ethics committee approval, 70 American Society of Anesthesiologists I–II patients scheduled for elective LH were enrolled prospectively. Standard anesthesia monitoring was supplemented with radial arterial cannulation to assess cardiac output via arterial waveform analysis. After anesthesia induction and tracheal intubation, patients were randomly divided into 2 groups: Group I (RM) and Group II (Control). Group I underwent multi-step RM twice intraoperatively. Hemodynamic parameters, arterial blood gases, and compliance measurements were recorded throughout surgery. Durations of surgery, anesthesia, and postanesthesia care unit stay were documented. Pulmonary complications were assessed on postoperative days 1 and 3.

**Results:**

Demographics and durations of anesthesia, surgery, and postanesthesia care unit stay were comparable between groups. Inotropic requirements and intraoperative fluid volumes were similar. Heart rate remained comparable; however, mean arterial pressure, cardiac index, and stroke volume index were significantly higher in Group I, while stroke volume variation, pulse pressure variation, and systemic vascular resistance index were significantly lower (*P* < .001). These hemodynamic changes were pronounced at peak RM pressure. Group I exhibited significantly higher dynamic lung compliance, PaO_2_, and PaO_2_/FiO_2_ ratios (*P* < .001). Overall, RM was associated with transient, hemodynamically tolerable changes alongside improved lung mechanics and oxygenation.

**Conclusion:**

Alveolar RM improved intraoperative lung compliance and oxygenation during LH without causing clinically significant hemodynamic instability. Incorporating RM into intraoperative management of laparoscopic procedures appears beneficial. These hemodynamic changes were transient and were not associated with an increased vasopressor requirement or clinically relevant hemodynamic instability.

## 1. Introduction

Laparoscopic hysterectomy (LH) is a minimally invasive surgical technique for uterine removal that offers several advantages over open surgery, including a smaller incision, reduced risk of adhesions, decreased analgesic requirement, shorter hospitalization, and faster recovery.^[1,2]^

However, pneumoperitoneum and the Trendelenburg position used during LH increase intra-abdominal and intrathoracic pressures. This elevates airway pressures and reduces lung compliance, thereby predisposing patients to intraoperative atelectasis.^[3,4]^ To mitigate this risk, lung-protective ventilation strategies: such as low tidal volume ventilation, individualized positive end-expiratory pressure (PEEP), and alveolar recruitment maneuvers (RM): are increasingly recommended.^[5,6]^

RM is designed to reopen collapsed alveoli by transiently applying elevated airway pressures, thereby improving lung compliance and preventing atelectasis during and after surgery. Despite these benefits, RM may trigger hemodynamic alterations, including a reduction in arterial pressure, cardiac output (CO), and systemic vascular resistance, as well as compensatory increases in heart rate (HR).^[7,8]^ Beyond conventional monitoring, minimally invasive techniques such as arterial waveform analysis allow for more precise assessment of these intraoperative hemodynamic effects and can guide perioperative management.^[9,10]^ Although the pulmonary benefits of RMs are well established, their intraoperative hemodynamic effects remain incompletely understood, particularly when assessed using minimally invasive CO monitoring during LH.

The primary aim of this prospective study was to investigate the impact of RM during LH on hemodynamic parameters: including CO, systemic vascular resistance, stroke volume (SV), stroke volume variation (SVV), and pulse pressure variation (PPV): as measured by arterial waveform analysis. The secondary aim was to evaluate its effects on dynamic lung compliance (Cdyn), oxygenation, and postoperative pulmonary complications.

## 2. Materials and methods

This prospective, randomized clinical trial was conducted at Balikesir University Faculty of Medicine following approval from the Institutional Ethics Committee (protocol no. 89017521/302.14.06/205138; date: December 7, 2022). All participants provided written informed consent prior to enrollment. The study complied with the principles of the Declaration of Helsinki and was registered at ClinicalTrials.gov (NCT07299396) on November 01, 2025. This study was conducted and reported according to the CONSORT (Consolidated Standards of Reporting Trials) guidelines.^[11,12]^

Sample size was calculated using G*Power software version 3.1.9.4, based on data from previous studies.^[9,10]^ A minimum of 32 patients per group was required to achieve 80% power at a 0.05 alpha error. To account for potential data loss, 35 patients were included in each group, resulting in a total of 70 participants.

Seventy female patients aged 18 to 70 years, classified as American Society of Anesthesiologists physical status I–II, scheduled for elective LH under general anesthesia, were recruited. Exclusion criteria included refusal to participate; invasive mechanical ventilation within the past month; chemotherapy or radiotherapy within the past 2 months; severe pulmonary disease (COPD, asthma, pulmonary infection, bronchiectasis, history of noninvasive ventilation, oxygen therapy, or steroid therapy for recent exacerbation); severe pulmonary hypertension; intracranial tumors; hemodynamic instability or shock; neuromuscular disorders; conditions increasing intracranial or intraocular pressure; atrial fibrillation; and body mass index (BMI) > 40.

Patients were randomized into 2 groups using a computer-generated randomization table: Group I (RM group, n = 35): received an alveolar RM intraoperatively. Group II (Control group, n = 35): did not receive RM.

All patients underwent preoperative evaluation including history, physical examination, and routine laboratory tests. Age, height, weight, and BMI were recorded. Standard intraoperative monitoring included electrocardiogram, pulse oximetry, noninvasive blood pressure, and esophageal temperature. Radial arterial cannulation was performed in all patients for invasive pressure monitoring and CO analysis using the ProAQT/PulsioFlex (Getinge, Germany) system.

Premedication was provided with intravenous midazolam (0.02 mg/kg). General anesthesia was induced with propofol (2 mg/kg), fentanyl (2 µg/kg), and rocuronium (0.6 mg/kg). Orotracheal intubation was performed with a 7.0 to 7.5 mm endotracheal tube. Ventilation was delivered using a Dräger Perseus A500 anesthesia workstation in Volume AutoFlow mode with the following parameters: tidal volume 6 to 8 mL/kg, respiratory rate 12 to 16 breaths/min, FiO_2_ 40% to 45%, PEEP 7 cmH_2_O, I:E ratio 1:2, and end-tidal carbon dioxide maintained at 35 to 45 mm Hg.

In Group I, RM was applied twice intraoperatively using the ventilator’s automated multi-step protocol:

First RM: Following intubation, before Trendelenburg positioning and CO_2_ insufflation.

Second RM: After CO_2_ deflation and the return to the supine position.

The RM was performed with a driving pressure of 10 to 12 cmH_2_O, a maximum PEEP of 20 cmH_2_O and a maximum inspiratory pressure of 35 cmH_2_O. PEEP was increased incrementally every 3 breaths until maximum pressure was achieved, then maintained for 6 breaths, and decreased stepwise. Each maneuver lasted approximately 2.5 to 3 minutes.

Hemodynamic variables: including mean arterial pressure (MAP), HR, cardiac index (CI), stroke volume index (SVI), SVV, PPV, and systemic vascular resistance index (SVRI): were recorded at baseline (T0) and at defined intraoperative time points. Cdyn and end-tidal carbon dioxide were also measured.

Measurements were obtained at: T0 (baseline prior to anesthesia), T1 (immediately following intubation), T2 (10 minutes post-intubation), T3 (before surgical positioning with first arterial blood gas sampling). In Group I, the first RM included measurements at T4.1 (before RM initiation), T4.2 (at peak airway pressure), and T4.3 (following RM completion with second arterial blood gas sampling). Group II maintained standard ventilation during this period. Following surgical positioning and pneumoperitoneum establishment at 15 mm Hg with Trendelenburg position, measurements were recorded from T5 (immediately after surgical commencement) through T10 throughout the intraoperative period. At T11 (following CO_2_ deflation and return to supine position), arterial blood gas sampling was performed. The second RM in Group I included measurements at T12.1 (before RM initiation), T12.2 (at peak airway pressure), and T12.3 (following RM completion with fourth arterial blood gas sampling). Final measurements were obtained at T13 immediately before extubation.

Durations of surgery, anesthesia, and postanesthesia care unit stay, the amount of intraoperative fluid and vasoactive drugs administered, and urine output were recorded. Postoperative assessments were performed on days 1 and 3 for signs of respiratory complications.

Data were analyzed using IBM SPSS Statistics version 25.0. Normality was tested with the Kolmogorov–Smirnov test. Continuous variables were expressed as mean ± standard deviation or median (min–max), and categorical variables as counts (n). Cross-group comparisons were performed using independent samples *t* test or Mann–Whitney *U* test as appropriate. Categorical data were compared with Pearson’s chi-square test. A *P*-value < .05 was considered statistically significant.

## 3. Results

Our study recruited 70 patients (35 in Group I (RM) and 35 in Group II (Control)). The 2 groups had similar demographic data and durations of anesthesia, surgery, and postanesthesia care unit stay (Table 1). Overall, MAP and CI decreased transiently during RMs, whereas Cdyn and oxygenation improved significantly, without clinically important hemodynamic instability.

**Table 1:** Demographic data and duration of anesthesia, surgery, and PACU.

	Group I (RM) (n = 35)	Group II (Control) (n = 35)	*P* value
Age (yr)	48.28 ± 8.39	49.89 ± 8.91	.442
Height (cm)	161.46 ± 5.16	160.14 ± 5.98	.329
Weight (kg)	71.11 ± 14.04	76.49 ± 13.73	.356
BMI (kg/m^2^)	27.30 ± 5.09	29.18 ± 4.59	.110
ASA I/II	8/27	7/28	.771
Anesthesia duration (min.)	117.31 ± 18.55	120.51 ± 31.49	.606
Surgery duration (min.)	97.77 ± 19.52	100.57 ± 31.56	.657
PACU duration (min.)	7.91 ± 2.28	8 ± 2.52	.882

Data are presented as mean ± standard deviation and number (n).

RM = recruitment maneuver, BMI = body mass index, ASA = American Society of Anesthesiologists, PACU = postanesthesia care unit.

Initial (T0) HR values were measured as 77.17 ± 12.49 in Group I and 83.14 ± 15.99 in Group II. HR values were similar between the groups at all time points (*P* < .001). In both groups, HR values after intubation (T1) were significantly higher compared to all other time points (*P* < .001). No significant changes in HR were observed during the RM periods.

Compared to initial values, MAP was significantly lower in Group I at T4.2, T5-T13, and in Group II at T2-T5, T12.1, and T12.3 (*P* = .016). Although baseline values were similar, MAP values at T4.2, T5-T9, and T11-T13 in Group I were lower than Group II (*P* = .016). During RM periods in Group I, MAP at T4.2 was significantly lower compared to T4.1 and T4.3 (*P* = .016), and values at T12.2 and T12.3 were lower than at T12.1 (*P* = .016).

Post-intubation CI values were 3.45 (2.06–5) in Group I and 3.58 (2.06–5.07) in Group II. CI values at T4.2 and T6 in Group I were significantly lower than those in Group II (*P* < .001). In both groups, CI values at all time points showed a significant decrease compared to T1 (*P* < .001). In Group I, during the first RM period, values at T4.2 and T4.3 were lower than at T4.1, and the value at T4.2 was also lower than T4.3 (*P* < .001). In the second RM period, values at T12.2 and T12.3 were found to be lower than at T12.1 (*P* < .001) (Fig. 1A).

**Figure 1.**
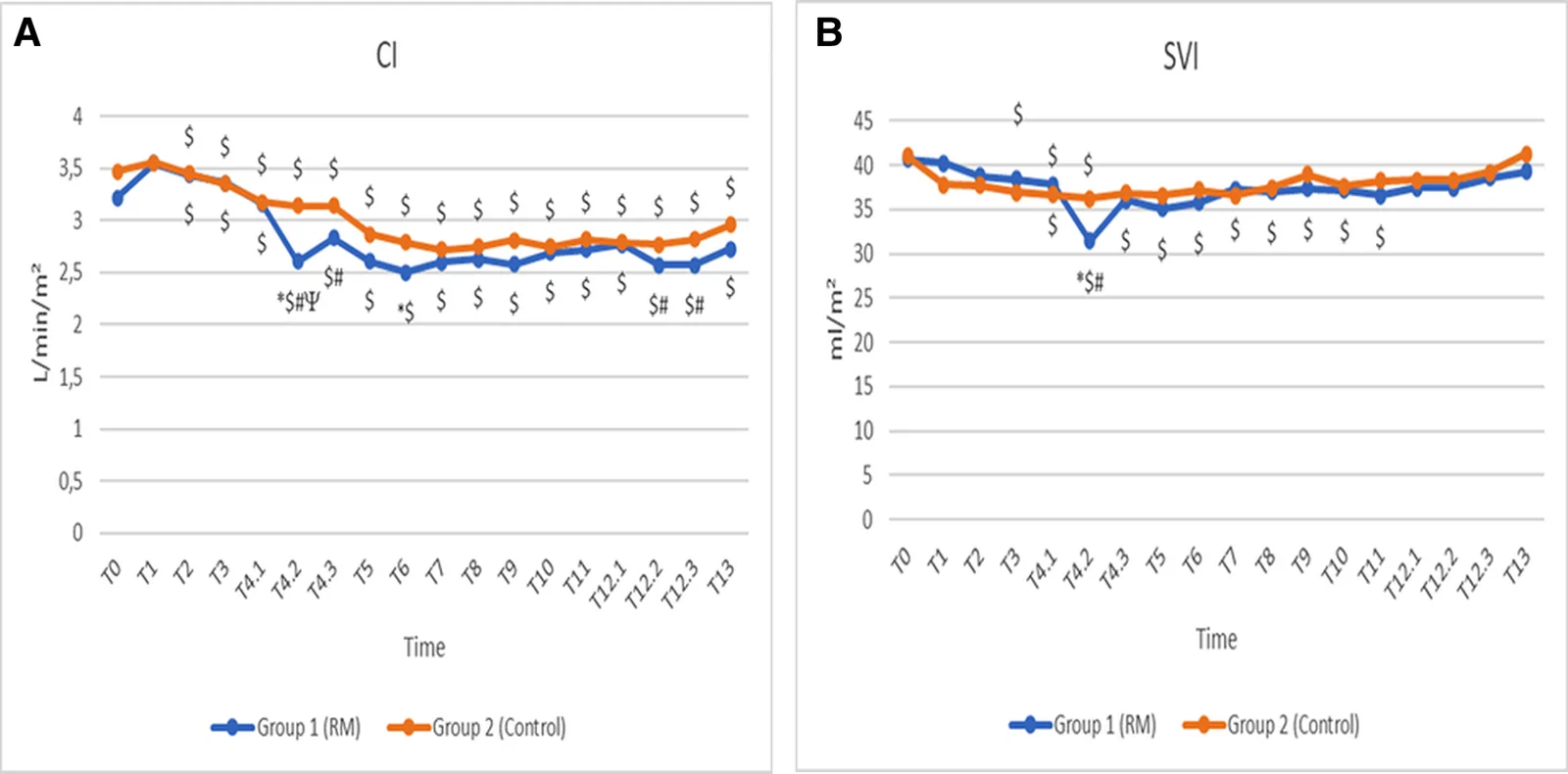
Cardiac index data of the groups. * Comparison between Group I and Group II, *P* < .05, $: Intragroup comparison for Group I (RM) and Group II (Control), *P* < .05 compared to baseline value, #,Ψ: Intragroup comparison for Group I (RM) during RM periods, *P* < .05. (B) Stroke volume index data of the groups.* Comparison between Group I and Group II, P < .05. $: Intragroup comparison for Group I (RM) and Group II (Control), *P* < .05 compared to baseline value. #,Ψ: Intragroup comparison for Group I (RM) during RM periods, *P* < .05.

SVI values measured after intubation were 40 (28–54) in Group I and 36 (13–56) in Group II. The value at T4.2 in Group I was significantly lower than Group II (*P* = .017). In Group I, during the first RM period, the value at T4.2 was lower than at T4.1 and T4.3 (*P* = .017) (Fig. 1B).

SVV values measured after intubation were 12 (2–21) in Group I and 12 (4–25) in Group II, and PPV values were 10 (4–24) and 11 (4–24) respectively. At T4.2, T8, and T12.2 in Group I, both SVV and PPV values were significantly higher compared to Group II (*P* < .001). During RM periods, SVV and PPV values at T4.2 were higher than those at T4.1 and T4.3 (*P* < .001), and values at T12.2 were higher compared to other RM time points (*P* < .001). (Figs. 2A and 2B).

**Figure 2.**
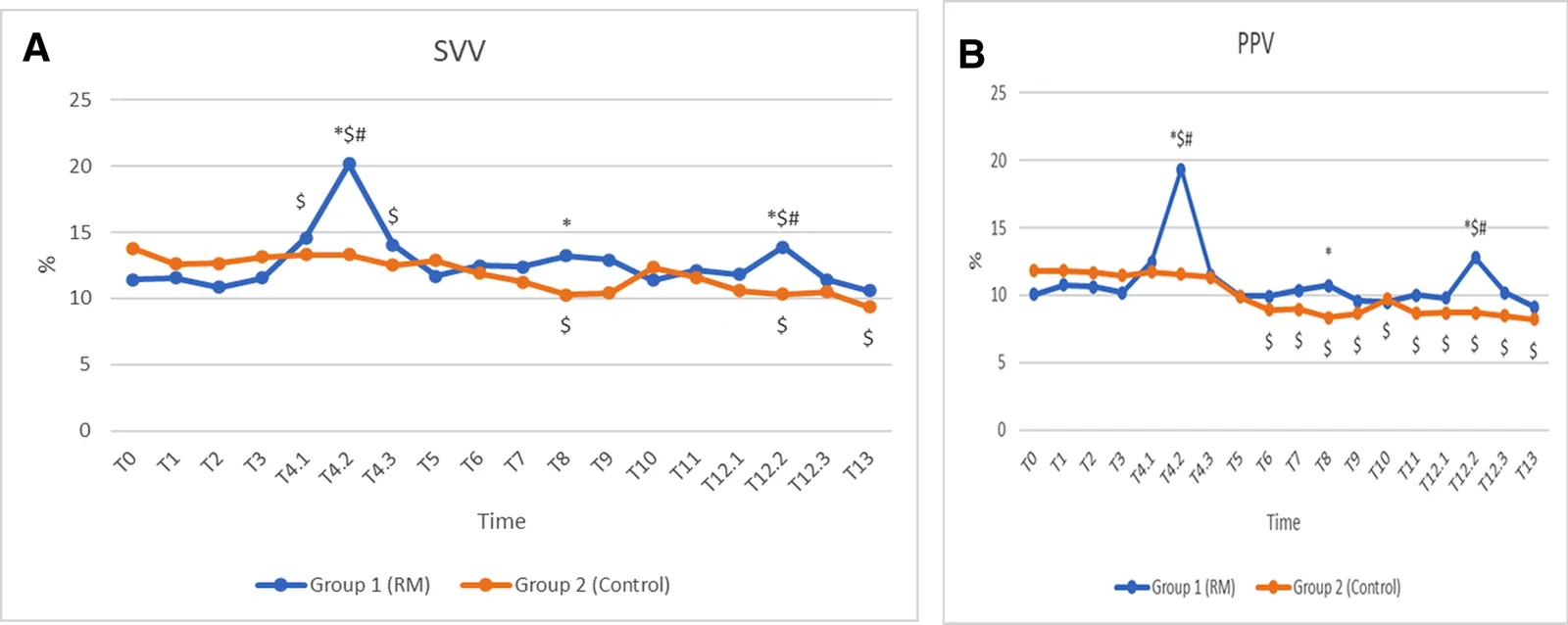
Stroke volume variation data of the groups. *: Comparison between Group I and Group II, *P* < .05. $: Intragroup comparison for Group I (RM) and Group II (Control), *P* < .05 compared to T1 value. #: Intragroup comparison for Group I (RM) during RM periods, *P* < .05. (B) Pulse pressure variation data of the groups. *: Comparison between Group I and Group II, *P* < .05. $: Intragroup comparison for Group I (RM) and Group II (Control), *P* < .05 compared to T1 value. #: Intragroup comparison for Group I (RM) during RM periods, *P* < .05.

Post-intubation SVRI values were 2424 (1373–3804) in Group I and 2126 (640–3880) in Group II. SVRI values at T4.2 and T4.3 in Group I were significantly higher than Group II (*P* = .048). In both groups, SVRI values at T5-T13 were significantly higher compared to T1 (*P* = .048). During RM periods in Group I, values at T4.2 and T4.3 were higher than T4.1, and values at T12.2 and T12.3 were higher than T12.1 (*P* = .048) (Fig. 3A).

**Figure 3.**
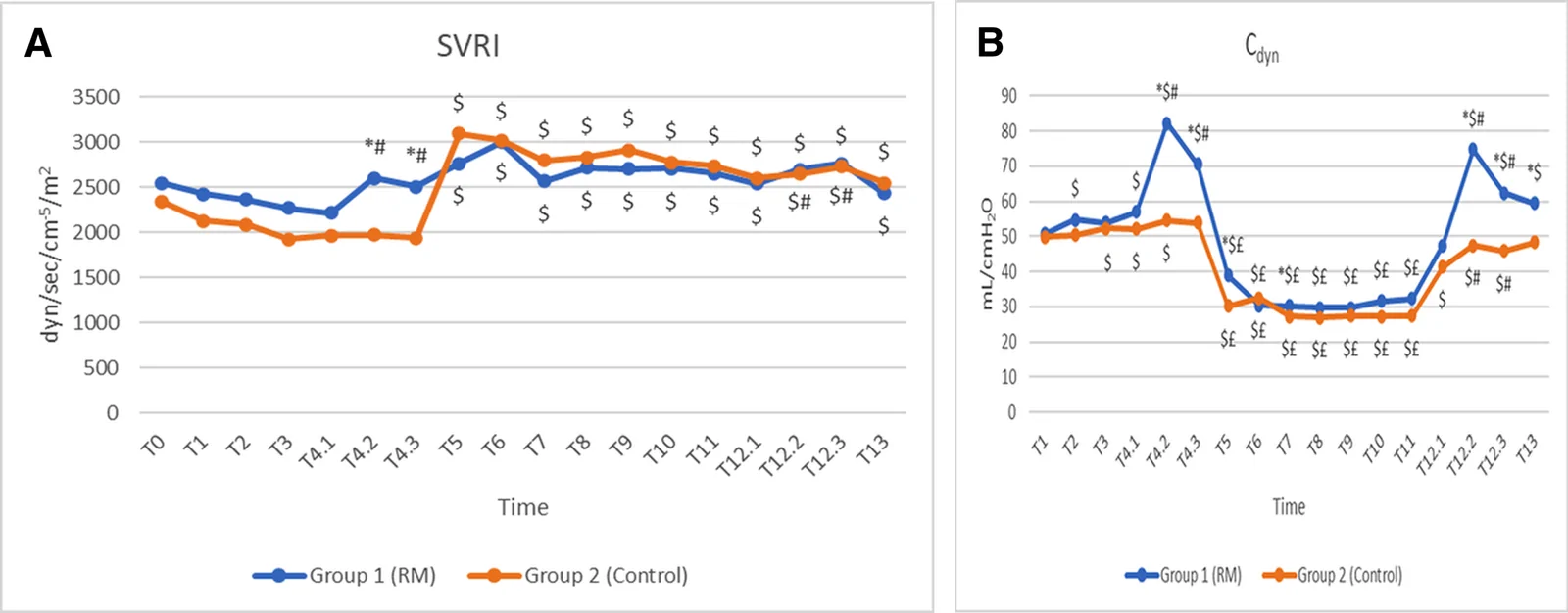
Systemic vascular resistance index data of the groups. *: Comparison between Group I and Group II, *P* < .05. $: Intragroup comparison for Group I (RM) and Group II (Control), *P* < .05 compared to T1 value, #: Intragroup comparison for Group I (RM) during RM periods and Group II (Control) at corresponding time points, *P* < .05, £: Intragroup comparison for Group I (RM) and Group II (Control) of the insufflation and Trendelenburg period compared to other measurement times, *P* < .05. (B) Dynamic compliance data of the groups. *: Comparison between Group I and Group II, *P* < .05. $: Intragroup comparison for Group I (RM) and Group II (Control), P < .05 compared to T1 value. #: Intragroup comparison for Group I (RM) during RM periods and Group II (Control) at corresponding time points, *P* < .05. £: Intragroup comparison for Group I (RM) and Group II (Control) of the insufflation and Trendelenburg period compared to other measurement times, *P* < .05.

Post-intubation Cdyn values were 49.80 (24.90–83.30) in Group I and 43.70 (31–86.30) in Group II. Cdyn values at T4.2, T4.3, T5, T7, T12.2, T12.3, and T13 in Group I were significantly higher than those in Group II (*P* < .001).

In Group I, Cdyn values increased at T2, T4.1–T4.3, and T12.2–T13 compared to T1, while they decreased at T5–T11 (*P* = .003). In Group II, an increase was observed at T3–T4.2, and a decrease was observed at T5–T12.3 (*P* = .003). During RM periods, in Group I, values at T4.2 and T4.3 were higher than T4.1, and values at T12.2 and T12.3 were higher than T12.1 (*P* = .003). In both groups, Cdyn values during pneumoperitoneum and Trendelenburg positioning (T5–T11) were significantly lower compared to other time points (*P* = .003) (Fig. 3B).

In the intergroup comparison, SaO_2_ values at the 1st blood gas were significantly higher in Group I compared to Group II (*P* = .04), while pH values at the 3rd blood gas were significantly lower in Group I (*P* = .04).

In the intragroup assessment, PaO_2_ values at the 2nd blood gas in Group I were significantly higher compared to the 1st blood gas (*P* = .04). In Group I, PaO_2_ and SaO_2_ values at the 4th blood gas showed a significant increase compared to the 3rd blood gas (*P* = .04). In Group II, pH and PaCO_2_ values at the 1st blood gas were significantly lower, while SaO_2_ values were significantly higher compared to the 3rd blood gas (*P* = .04).

While no significant difference was observed between the groups in terms of PaO_2_/FiO_2_ ratios (*P* = .04), in Group I, the values at the 2nd blood gas were significantly higher than the 1st blood gas, and the values at the 4th blood gas were significantly higher than the 3rd blood gas (*P* = .04). No statistically significant change was detected in these ratios in Group II (*P* = .04) (Table 2).

**Table 2:** Arterial blood gas values.

		Group I (RM) (n = 35)	Group II (Control) (n = 35)
PH	*1.BG*	7.40 ± 0.03	7.30 ± 0.49‡
•	*2.BG*	7.39 ± 0.03	
•	*3.BG*	7.32 ± 0.04*	7.36 ± 0.51
•	*4.BG*	7.33 ± 0.56	
PaCO_2_	*1.BG*	37.10 ± 3.51	39.42 ± 7.86‡
•	*2.BG*	37.48 ± 3.65	
•	*3.BG*	42.48 ± 4.55	41.07 ± 4.80
•	*4.BG*	42.18 ± 7.67	
PaO_2_	*1.BG*	137.36 ± 57.80	131.13 ± 49.42
•	*2.BG*	170.55 ± 65.87†	
•	*3.BG*	148.24 ± 45.26	129.92 ± 38.64
•	*4.BG*	179.86 ± 55.92†	
SaO_2_	*1.BG*	99.51 ± 1.07*	98.55 ± 1.46‡
•	*2.BG*	99.29 ± 2.39	
•	*3.BG*	98.69 ± 1.40	97.99 ± 2.01
•	*4.BG*	99.20 ± 1.20†	
PaO_2_/FiO_2_	*1.BG*	318.91 ± 126.86	374.28 ± 113.83
*•*	*2.BG*	420 ± 140.02†	
*•*	*3.BG*	395 ± 101.06	345.71 ± 107.17
*•*	*4.BG*	462.22 ± 119.76†	

Data are presented as mean ± standard deviation.

* Comparison between Group I and Group II, *P* < .05.

† Comparison in Group I (RM) before and after RM, *P* < .05.

‡ Intragroup comparison in Group II (Control), *P* < .05.

The amounts of intravenous fluids administered intraoperatively and urine output were similar between groups. Positive inotropic drugs were administered to 7 patients in Group I (RM) and 2 patients in Group II; however, this difference was not statistically significant. Atropine was not administered to any patient.

In the evaluation performed on postoperative days 1 and 3, no signs suggesting respiratory tract infection, bronchospasm, pleural effusion, atelectasis, or respiratory failure were found in any patient.

## 4. Discussion

We investigated the effects of RM during the intraoperative period in laparoscopic hysterectomies and found that RM caused a decrease in MAP without inducing any change in HR. It also led to a reduction in CI and SVI, as well as an increase in SVV and PPV, but only during RM application. However, these effects were restricted and did not necessitate additional vasopressor or fluid requirements from a clinical perspective. Furthermore, RM application was associated with an increase in lung compliance and an improvement in oxygenation. These findings suggest that the observed hemodynamic changes were transient in nature and did not translate into clinically meaningful instability requiring additional intervention.

The risk of atelectasis developing under general anesthesia becomes more pronounced with the Trendelenburg position, obesity, and laparoscopic/robotic/thoracic/cardiac surgery, leading to an increased risk of postoperative pulmonary complications.^[1,2]^ To mitigate this risk, the application of intraoperative lung-protective ventilation strategies: such as low tidal volume, personalized PEEP, and RM: is recommended.^[2]^ These strategies aim to avoid lung overdistension, prevent cyclic opening and closing injury, and refrain from the use of high FiO_2_. Our study examined the effects of RM, applied to prevent atelectasis in Trendelenburg-positioned LH, on cardiac and respiratory parameters.

RM is effective in preventing atelectasis associated with general anesthesia and can be applied via a ventilator using manual techniques, sustained increases in airway pressure, or stepwise pressure increases.^[5]^ Manual RM (bag squeezing) has been abandoned due to safety concerns and the risk of alveolar recollapse.^[5,13]^ RM studies have been conducted with varying methods, timing, pressure levels, and frequencies.^[14–19]^ This diversity stems from safety concerns, the pursuit of efficacy, and patient variability. Recent studies show that a driving pressure below 13 cmH_2_O reduces the risk of postoperative pulmonary complications.^[20–23]^ In our study, RM was applied using the ventilator’s automated multi-step RM mode (pressure-controlled mode, stepwise increase) in patients with BMI < 40: with settings as followed: maximum PEEP of 20 cmH_2_O, maximum peak inspiratory pressure of 35 cmH_2_O, and driving pressure of 10 to 12 cmH_2_O; the initial and final PEEP was set at 7 cmH_2_O. This approach utilized the effective lowest peak pressure and PEEP values with a driving pressure unlikely to cause alveolar damage, applied over the minimum effective number of breaths.

Whalen et al reported that in laparoscopic surgery, MAP and HR measurements in patients receiving 12 cmH_2_O PEEP and RM were similar to those receiving only 4 cmH_2_O PEEP. They noted that while patients receiving the RM did not require vasopressors during the maneuver itself, they consumed more vasopressors throughout the surgery.^[24]^ Similarly, numerous studies have shown that RM has no significant effect on HR.^[7,25,26]^ We concluded that RM did not cause a significant change in HR. In patients receiving RM, a decrease in MAP was statistically significant, particularly at peak PEEP levels and at the end of the maneuver; however, this effect was transient and did not necessitate additional intervention. Vasopressor requirement was observed in 7 patients in the RM group and 2 patients in the control group. Although MAP was significantly lower in the RM group throughout the surgery, it did not lead to a significant increase in vasopressor requirement.

In a study utilizing pulse contour analysis, Toth et al observed a strong negative correlation between increased central venous pressure during RM and CO, along with a significant decrease in CO.^[8]^ Nielsen et al also detected a significant decrease in CO with RM using a similar method.^[27]^ Monitoring cardiac functions solely with MAP and HR may lead to an overlook of the hemodynamic effects of RM.^[7]^ Similar to Toth et al we monitored the instantaneous effects of increased transpulmonary pressure on the heart during RM using pulse contour analysis. Increased transpulmonary pressure from RM compresses the right atrium, raising central venous pressure, and the reduction in left ventricular preload leads to a decrease in CO.^[25]^ In the RM group, CI values were significantly lower than control when peak PEEP was reached. Between the first and second RM, CI values were lower only at the 10-minute post-RM measure in the RM group. We posit that the application of Trendelenburg position and pneumoperitoneum in all patients limited the RM-induced decrease in CI. Due to the absence of a similar effect on transpulmonary pressure between the groups after the second RM, the decrease in CI during the second RM persisted in measurements taken at the end of RM and before extubation.

The degree of decrease in CO caused by RM is closely related to the patient’s preload (volemic status). Post-intubation SVV and PPV values were similar between groups, this was probably due to the preoperative ~10-hour fasting period which was also similar in all patients. During the first RM, when peak PEEP was reached, SVV and PPV values increased significantly compared to the control group; this increase was not observed during the second RM. We believe that the administered fluid throughout the surgery attenuated the intensity of the SVV and PPV response during the second RM.

Monge et al investigated the respiratory/hemodynamic effects of RM with stepwise PEEP increase and detected an increase in SVRI during the PEEP increase phase and a significant rise in SVRI at peak PEEP compared to baseline. It was reported that SVRI returned to baseline levels upon PEEP reduction.^[28]^ Similarly, in our study, SVRI measured at peak PEEP and at the end of RM was significantly higher than at the start of the maneuver.

It has been demonstrated that arterial blood gas values return to preoperative levels by the 24th postoperative hour.^[29,30]^ In our study, RM increased PaO_2_ and the PaO_2_/FiO_2_ ratio but did not produce a significant difference in PaCO_2_. Whalen et al evaluated the effect of RM on arterial oxygenation in laparoscopic bariatric surgery; they reported that PaO_2_ and PaO_2_/FiO_2_ ratios, as well as compliance, were significantly higher in the RM group, while PaCO_2_ values were similar.^[24]^ Cdyn values remained higher for 40 minutes after the first RM and until extubation after the second RM in patients who received RM, compared to those who did not. In the RM group, PaO_2_ and the PaO_2_/FiO_2_ ratio improved. The applied pneumoperitoneum and Trendelenburg position caused an increase in airway pressure/resistance and a decrease in lung compliance in both groups.

Our study has several limitations. Only female patients undergoing LH without respiratory system disease were included. The absence of bleeding/fluid management issues during surgery limits the broader interpretation of RM’s effects in such scenarios. The lack of objective methods, such as ultrasonography, for intraoperative/postoperative atelectasis assessment challenges the objectivity of the data. In addition, this was a single-center study with a relatively small sample size, which may limit the generalizability of the findings.

## 5. Conclusion

Alveolar RMs improved intraoperative lung compliance and oxygenation during LH without causing clinically relevant hemodynamic instability. Although transient reductions in arterial pressure and CI occurred during RMs, these changes did not increase vasopressor requirements. These findings support the use of RMs as part of lung-protective ventilation strategies in appropriately selected patients undergoing laparoscopic surgery.

## Acknowledgments

This research received no external funding. The authors declare no conflict of interest. Ethical approval was granted by the Ethics Committee of Balikesir University Faculty of Medicine (protocol no. 89017521/302.14.06/205138; approval date: December 7, 2022), and written informed consent was obtained from all participants. No individuals other than the listed authors contributed to this work.

## Author contributions

**Conceptualization:** Hasan Duman, Ozlem Sagir.

**Data curation:** Hasan Duman, Ebru Duman, Hafize Fisun Demir, Nazan Kocaoglu Bugra, Serkan Uckun, Ozlem Sagir.

**Formal analysis:** Hasan Duman, Ebru Duman, Hafize Fisun Demir, Fatih Ugun, Nazan Kocaoglu Bugra, Serkan Uckun, Ozlem Sagir.

**Funding acquisition:** Hasan Duman, Ebru Duman, Hafize Fisun Demir, Fatih Ugun, Nazan Kocaoglu Bugra, Serkan Uckun, Ozlem Sagir.

**Investigation:** Hasan Duman, Ebru Duman, Hafize Fisun Demir, Fatih Ugun, Nazan Kocaoglu Bugra, Serkan Uckun, Ozlem Sagir.

**Methodology:** Hasan Duman, Ebru Duman, Hafize Fisun Demir, Fatih Ugun, Nazan Kocaoglu Bugra, Serkan Uckun, Ozlem Sagir.

**Project administration:** Hasan Duman, Ebru Duman, Hafize Fisun Demir, Fatih Ugun, Nazan Kocaoglu Bugra, Serkan Uckun, Ozlem Sagir.

**Resources:** Hasan Duman, Ebru Duman, Hafize Fisun Demir, Fatih Ugun, Nazan Kocaoglu Bugra, Serkan Uckun, Ozlem Sagir.

**Software:** Hasan Duman, Ebru Duman, Hafize Fisun Demir, Fatih Ugun, Nazan Kocaoglu Bugra, Serkan Uckun, Ozlem Sagir.

**Supervision:** Hasan Duman, Ebru Duman, Hafize Fisun Demir, Fatih Ugun, Nazan Kocaoglu Bugra, Serkan Uckun, Ozlem Sagir.

**Validation:** Hasan Duman, Ebru Duman, Hafize Fisun Demir, Fatih Ugun, Nazan Kocaoglu Bugra, Serkan Uckun, Ozlem Sagir.

**Visualization:** Hasan Duman, Ebru Duman, Hafize Fisun Demir, Fatih Ugun, Nazan Kocaoglu Bugra, Serkan Uckun, Ozlem Sagir.

**Writing – original draft:** Hasan Duman, Ebru Duman, Hafize Fisun Demir, Fatih Ugun, Nazan Kocaoglu Bugra, Serkan Uckun, Ozlem Sagir.

**Writing – review & editing:** Hasan Duman, Ebru Duman, Hafize Fisun Demir, Fatih Ugun, Nazan Kocaoglu Bugra, Serkan Uckun, Ozlem Sagir.
